# Comparison Study of Four Extraction Methods Combined with PCR and LAMP for Feline *Tritrichomonas foetus* Detection in Fecal Samples

**DOI:** 10.3390/pathogens11050604

**Published:** 2022-05-22

**Authors:** Joanna Dąbrowska, Jacek Karamon, Maciej Kochanowski, Jacek Sroka, Jolanta Zdybel, Tomasz Cencek

**Affiliations:** Department of Parasitology and Invasive Diseases, National Veterinary Research Institute, Partyzantów Avenue 57, 24-100 Puławy, Poland; j.karamon@piwet.pulawy.pl (J.K.); maciej.kochanowski@piwet.pulawy.pl (M.K.); jacek.sroka@piwet.pulawy.pl (J.S.); j.zdybel@piwet.pulawy.pl (J.Z.); tcencek@piwet.pulawy.pl (T.C.)

**Keywords:** *Tritrichomonas foetus*, cat, DNA extraction methods

## Abstract

Feline trichomonosis occurs worldwide, with gastrointestinal symptoms such as chronic large-bowel diarrhea and abdominal pain. The inclusion of molecular methods in diagnostic and epidemiological studies has necessitated an effective method for extracting DNA from feces. We tested four extraction commercial kits: ZR Fecal DNA MiniPrep (50 preps) (Zymo Research, Irvine, CA, USA), QIAamp^®^ DNA Stool Mini Kit (Qiagen Inc., Valencia, CA, USA), UltraClean Fecal DNA Kit (50 preps) (MO BIO, San Diego, CA, USA), and Sherlock AX/100 isolations (A&A Biotechnology, Gdynia, Poland). We assessed the sensitivity of detection of *Tritrichomonas foetus* in spiked fecal samples for the four kits combined with two molecular assays: PCR and LAMP. The extraction efficacy was quantified using defined aliquots of fecal samples spiked with 5 μL of suspensions containing serial dilutions of trophozoites (0.1; 1; 10; 100; 1000; 10,000), with six replicates for each concentration. In our study, we proved that the ZR Fecal DNA MiniPrep (50 preps) kit combined with LAMP and PCR had the highest efficiency among all the compared methods for the detection of feline *T. foetus* from fecal samples.

## 1. Introduction

*Tritrichomonas foetus* is a protozoan parasite living in the gastrointestinal tracts of cats. The parasite colonizes the ileum, caecum, and colon in close proximity to the mucosal surface. The disease it causes, trichomonosis, results in symptoms, such as chronic diarrhea, often associated with blood and mucus, which can lead to dehydration [1]. Furthermore, cats also suffer from tenesmus, flatulence, and anal irritation. Trichomonads are transmitted by the oral–fecal route during the common sharing of a litter box or mutual grooming [2]. Most of the affected cats come from rescue shelters or pedigree-breeding colonies. Feline trichomonosis has been reported worldwide in animals, with a prevalence of infection of 2–59% [3,4,5]. The disease has been diagnosed in both pure-bred and non-bred cats and in both males and females. Although, according to Stockdale et al. [6] and Dąbrowska et al. [7], cats less than one year of age are more susceptible to the disease, animals of all ages may become ill [8,9,10]. Different methods for *T. foetus* identification exist, and the most popular is microscopic examination. Trophozoites of *T. foetus* may be detected via direct fecal smears, wet mounts, or in cell culture under light microscopy. However, the motile form of parasites may be mistaken for other feline parasites, e.g., *Pentatrichomonas hominis* or *Giardia* spp [11]. Therefore, polymerase chain reactions have become the most efficient tools for detecting *T. foetus* in cats. These methods are known to have much better sensitivity and specificity than microscopic examinations and allow for the identification of parasite DNA even from dead cells [12]. Furthermore, although less affordable for large-scale sampling, PCR is usually more specific and allows downstream applications compared to LAMP, e.g., definitive species determination [13].

LAMP methods have recently been introduced to trichomonosis diagnosis because they are simple to perform, and they have become an alternative to conventional PCR. Furthermore, LAMP is a molecular method, which in comparison to the PCR is cheaper and faster [14,15]. However, the molecular detection of feline *T. foetus* in fecal specimens has limitations caused by the poor recovery of DNA and by the presence of amplification inhibitors [16]. Feces are a very complex mixture of organic and inorganic compounds that can significantly inhibit PCRs [17]. Therefore, one of the most crucial steps of testing samples from cats is finding an effective and appropriate kit for DNA isolation. Many authors [18,19,20,21,22] conducted similar studies with different parasites with molecular methods (Supplementary File S1.1).

Although studies comparing methods for directly *T. foetus* extraction from feces have already been performed by Stauffer [23], the aim of this study was to compare four commonly used DNA isolation kits. Here, we evaluate their influence on the performance of PCR according to Felleisen et al. [24] and LAMP according to Dąbrowska et al. [25] for the detection of *T. foetus* in feline feces. Furthermore, we used a Sherlock AX/100 isolations (A&A Biotechnology, Gdynia, Poland) as one of the compared kits.

## 2. Results

### Influence of DNA Extraction Methods on the Performance of Molecular Assays

The Z-kit combined with LAMP and PCR demonstrated the best performance among all the assays. This combination allowed the detection of the DNA of *T. foetus* from one cell of the parasite with 100% sensitivity. Lower performance for both molecular methods was observed in the case of extraction by Q-kit, which, nevertheless, allows for the detection of one cell of the parasite. However, LAMP combined with Q-kit exhibited two times more efficiency than PCR combined with the Q-kit.

The other two methods, S-LAMP and U-LAMP, were able to detect 10 and 100 trichomonad cells, respectively (Supplementary File S1.1). However, when using S-PCR, a higher percentage of positive results was found (50%) than in the case of U-PCR (16.6%). Detailed data with the percentage of positive results obtained using both assays combined with the four extraction methods are shown in Table 1.

**Figure 1:**
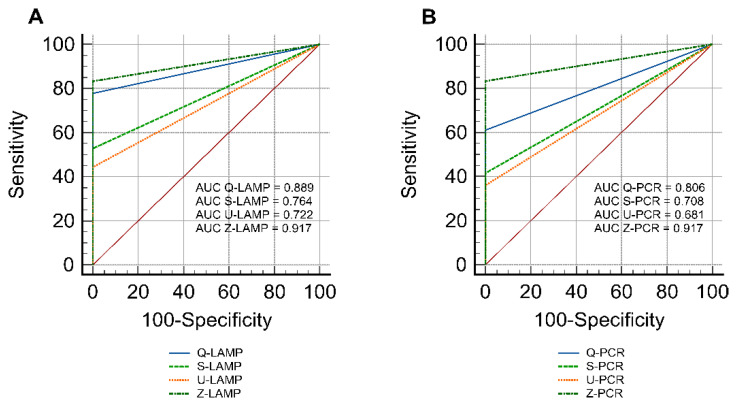
Receiver operating characteristic curve (ROC) plots with calculated Area Under the Curve (AUC) for the LAMPs and PCRs are shown in (**A**,**B**), respectively. The fecal samples used to validate the assays (spiked samples *n* = 36, and non-spiked samples *n* = 6) were subjected to this analysis. The MedCalc software (MedCalc Software Ltd., ver. 19.3, Mariakerke, Belgium) was used to perform calculations and plotting.

The calculated area under the ROC curve (AUC) was highest for the Z-kit combined with LAMP and PCR (0.917), indicating that this method showed the highest accuracy. A slightly lower AUC value was calculated for Q-LAMP (0.889). Other variants had the lower accuracy as follows: Q-PCR (0.806), S-LAMP (0.764), U-LAMP (0.722), S-PCR (0.708), and U-PCR (0.681) (Figure 1).

Statistically significant differences between the performance of the following variants of assays were observed: Z-LAMP vs. U-PCR and Z-PCR vs. U-PCR.

Detailed results for the statistical comparisons are shown in Supplementary File S1.2, with the percentages of positive results at all the spiking levels obtained with the four extraction methods combined with the PCR and LAMP assays.

## 3. Discussion

This study evaluated the performance of the combination of four commercially available extraction kits together with two different molecular assays for the detection of *T. foetus* DNA in spiked feline fecal samples. The best results were obtained with the Z-kit combined with LAMP and PCR, which was able to detect one cell of *T. foetus*. The Q-kit combined with both molecular methods also had high efficiency. However, the number of positive results obtained with Q-LAMP was higher than that obtained with Q-PCR at the same spiking level. Similarly, U-LAMP and S-LAMP were more sensitive than the PCRs. Based on these results, we assume that the kits combined with LAMP are able to detect low concentrations of *T. foetus* DNA due to high sensitivity and rapidity and, hence, can be applied even by non-specialists without the need for elaborate lab equipment. Furthermore, according to Dąbrowska et al. [25], LAMP and PCR combined with ZR Fecal DNA MiniPrep gave negative results in specificity reaction with other close related microorganisms, which indicates their diagnostic effectiveness in trichomonosis.

Similarly, Stauffer et al. [23] found that the ZR Fecal DNA MiniPrep (50 preps) Zymo Research, Irvine, CA kit had the highest efficiency in DNA isolation among all the compared methods, with a sensitivity of 10 *T. foetus* organisms per 100 mg of feces and with 10 trophozoites per 150 mg. However, even though a relatively high performance was reported by Stauffer with a single-tube nested PCR, our data provide evidence for the efficiency of the Z-kit combined with PCR according to Felleisein et al. [24] and LAMP according to Dąbrowska et al. [25]. 

Similar comparative studies with other parasites were also conducted. Govic et al. [19] proved that the ZR Fecal DNA MiniPrep method had higher efficiency than the NucliSens^®^ easyMAG^®^ (EM) system in coprological diagnosis of cryptosporidiosis in human fecal samples. Furthermore, Maksimov et al. [26] reported the highest sensitivity among four commercially available kits of ZR Fecal DNA MiniPrep™ combined with qPCR in the identification of the DNA of *Echinococcus multilocularis* in spiked fecal samples. Additionally, Yoshikawa et al. [20] also proved that, among five compared methods, ZR Fecal DNA MiniPrep was the most useful in the isolation of DNA of *Blastocystis* sp. from fresh fecal samples, with 94% positive results. It is worth noting that ZR Fecal DNA MiniPrep™ had a significantly higher ability for extracting DNA than the other isolation methods. In our comparative studies, we used Sherlock AX/100 isolation (A&A Biotechnology, Gdynia, Poland) for DNA extraction from materials with trace DNA contents. This method was chosen because of its versatility in extracting good-quality DNA from environmental matrices such as blood and saliva stains, hair, fur, tissue preserved in paraffin and formalin, fresh tissue, and fresh and frozen blood. This kit has surprisingly relatively high sensitivity in DNA extraction and, combined with LAMP, was already able to detect 10 parasites in 16.6% of results. Furthermore, Sherlock AX was relatively inexpensive in comparison with the other kits. Another advantage of this method is that DNA is precipitated after elution and then suspended in a low volume of water; thus, the concentration of the final product is higher.

## 4. Material and Methods

### 4.1. T. foetus Cells

Trophozoites of the feline *T. foetus* (reference strain ATCC 30924) were cultivated in vitro in InPouch^®^ TF-Feline (Biomed Diagnostics, White City, OR, USA) and incubated at 37 °C for 48 h. The number of parasites was determined microscopically using a Neubauer counting chamber (American Optical Company, Buffalo, NY, USA).

Aliquots of suspensions containing serial 1:10 dilutions of cultivated trophozoites from the feline *T. foetus* (previously disrupted by vortexing with glass beads for 10 min and sonification in ice for 30 s) were prepared for further experiments [25].

### 4.2. Feline Fecal Samples

Fecal samples were obtained from a clinically healthy cat (confirmed by real-time PCR according to Frey et al. [27]). To compare the DNA isolation methods, samples with the following weights of feces were prepared: 145 mg (ZR Fecal DNA MiniPrep™, Zymo Research, Freiburg, Germany), 245 mg (UltraClean^®^ Fecal DNA Isolation Kit, MO BIO Laboratories Inc., Carlsbad, CA, USA), 215 mg (QIAamp^®^ DNA Stool Mini Kit (50), Qiagen, Hilden, Germany), and 15 mg (Sherlock AX/100 isolations, A&A Biotechnology, Gdynia, Poland). In order to maintain the weight recommended by the manufacturer, we lowered the weight of the fecal samples by 5 mg.

### 4.3. Spiking of Fecal Samples with T. foetus Trophozoites

Defined aliquots of fecal samples were spiked with 5 μL of suspensions containing serial dilutions of prepared *feline T. foetus* trophozoites (0.1; 1; 10; 100; 1000; 10,000), with 6 replicates for each concentration. The total number of samples was 336 (294 spiked samples for each kit evaluated by two molecular methods and 42 samples without trophozoites in 6 repetitions).

### 4.4. DNA Extraction

The study was conducted on all the spiked fecal samples with the following four commercial DNA extraction kits which were evaluated:

Q-kit: QIAamp^®^ DNA Stool Mini Kit (Qiagen Inc., Valencia, CA, USA);

U-kit: UltraClean Fecal DNA Kit (50 preps) (MO BIO, San Diego, CA, USA);

Z-kit: ZR Fecal DNA MiniPrep (50 preps) (Zymo Research, Irvine, CA, USA);

S-kit: Sherlock AX/100 isolations (A&A Biotechnology, Gdynia, Poland).

All the kits were used according to the manufacturer′s recommendations without modifications. The characteristics of the four methods tested are summarized in Table 2.

### 4.5. Molecular Analysis

#### 4.5.1. Positive Controls

DNA extraction from *T. foetus* (reference strain ATCC 30924) cultures was performed with the DNeasy^®^ Blood and Tissue Kit (Qiagen, Hilden, Germany) using the protocol for cultured cells and used as a positive control.

#### 4.5.2. PCR

PCR was performed on all the extracted samples to detect the target genes, the 5.8S rRNA gene and the flanking internal transcribed spacer regions ITS1 and ITS2. The sequences were amplified based on a method previously described [24]. The PCR amplification was performed in a 50 µL reaction mixture, including 1 pmol of each primer (TRF3 and TRF4), 200 µL of each dNTP (Fermentas, Baden-Wurttemberg, Germany), 250 U Taq polymerase (Qiagen, Hilden, Germany), 5 µL 10× concentrated PCR buffer (Qiagen, Hilden, Germany), 28.6 µL of DNAse-free water (Fermentas, Baden-Wurttemberg, Germany), and 1 µL DNA. Cycling conditions involved an initial 30 s denaturation of 94 °C, annealing at 67 °C for 30 s, and extension at 72 °C for 90 s. Following 40 cycles, a final extension step of 15 min at 72 °C was added. For the detection, 5 µL of PCR product was electrophoresed on 2% agarose gels.

#### 4.5.3. LAMP

For the detection of the β-tubulin gene sequence of *T. foetus*, the LAMP procedure was performed as previously described [25]. In brief, the reaction was performed in 15 µL total volume and included 7.5 µL of Isothermal Mastermix (OptiGene, Horsham, UK), 1 µL of each primer (20 pmol TF-βtub-FIB/TF-βtub-BIP and 5 pmol TF-βtub-F3/TF-βtub-B3), 1.5 µL of PCR-grade H2O (Qiagen, Hilden, Germany), and 2 µL of the DNA template. Amplification was carried out in a Biometra thermocycler (Gottingen, Germany) at 65 °C for 1 h. After amplification, 1 µL 1:10 Sybr^®^ Green (10,000× concentrated in DMSO) (SYBR^®^ Green I Nucleic Acid Gel Stain, Invitrogen, Sydney, Australia) was added to each LAMP product. The results were assessed based on color changes (a positive result was considered yellow-greenish and a negative was a clear orange color).

#### 4.5.4. Statistical Analysis

The MedCalc software (MedCalc Software Ltd., ver. 19.3, Mariakerke, Belgium) was used to perform the ROC analysis and calculate the area under the ROC curve (AUC). This analysis was performed to evaluate the accuracy of all assays.

Two-tailed Fisher′s exact test with Bonferroni correction (*p* < 0.0018) (https://www.graphpad.com/quickcalcs/contingency1, accessed on 1 February 2022) was calculated to analyze the differences in *T. foetus* detection using the evaluated extraction methods and assays.

## 5. Conclusions

In conclusion, it was determined that, of the four methods, the ZR kit was best suited for the extraction of total DNA from feline fecal samples. We also established that this kit efficiently extracted DNA and facilitated the amplification of targets by LAMP and conventional PCR. However, we strongly recommend using ZR Fecal MiniPrep combined with LAMP due to the general highest sensitivity among all the compared methods.

## Figures and Tables

**Table 1:** Percentage of positive results at all spiking levels obtained with four extraction methods combined with PCR and LAMP assays.

Suspensions of *T. foetus* Cells	Z (*n* = 6)		U (*n* = 6)		Q (*n* = 6)		S (*n* = 6)	
	PCR	LAMP	PCR	LAMP	PCR	LAMP	PCR	LAMP
10,000	100%	100%	100%	100%	100%	100%	100%	100%
1000	100%	100%	100%	100%	100%	100%	100%	100%
100	100%	100%	16.6%	33.3%	100%	100%	50%	100%
10	100%	100%	n.d	n.d	33.3%	100%	n.d	16.6%
1	100%	100%	n.d	n.d	33.3%	66.6%	n.d	n.d
0.1	n.d	n.d	n.d	n.d	n.d	n.d	n.d	n.d

**Table 2:** Overview of tested DNA Isolation Kits.

Full Name of the Kit	Manufacturers Details	Kit Name Abbreviation	Recommended Sample Starting Amount	Extraction Method	Elution Volume (µL)
QIAamp^®^ DNA Stool Mini Kit	Qiagen Inc., Valencia, CA, USA	Q	180–220 mg	Manual	200
UltraClean Fecal DNA Kit (50 preps)	MO BIO, San Diego, CA, USA	U	250 mg	Manual	50
ZR Fecal DNA MiniPrep (50 preps)	Zymo Research, Irvine, CA, USA	Z	150 mg	Manual	100
Sherlock AX/100 isolations	A&A Biotechnology, Gdynia, Poland	S	10–20 mg	Manual	350

## Data Availability

Not applicable.

## Supplementary Materials

The following supporting information can be downloaded at: https://www.mdpi.com/article/10.3390/pathogens11050604/s1, Table: Supplementary File S1.1: Performance of different extraction kits combined with molecular methods for protozoan parasites identification in feces; Supplementary File S1.2: Detailed results of PCR assays and LAMP assays with each isolation method; Supplementary File S1.3: The table shows statistical comparison results using Fisher′s exact test with Bonferroni correction. Statistically significant differences (*p* < 0.0018) are highlighted in yellow.
