# Supplementary material for: Comparison Study of Four Extraction Methods Combined with PCR and LAMP for Feline Tritrichomonas foetus Detection in Fecal Samples
Source: Pathogens. 2022 May 22;11(5):604. doi: 10.3390/pathogens11050604 (PMC9143749; doi:10.3390/pathogens11050604)
Supplement: Supplementary file 1 [file pathogens-11-00604-s001.zip › Supplementary Materials/S1.1..pdf]

**Protozoan parasite**	**Commercial kit**	**Molecular method**	**Positive results**	**References**
Cryptosporidium spp	QIAamp DNA stool mini kit (Qiagen Hilden, Germany)	Nested PCR	93.8% (n=32)	[18]
Giardia duodenalis	Wizard magnetic DNA purification system (Promega Corporation, Madison, WI, USA)	Real-time PCR	100% (n=47)	
Entamoeba spp	PowerFecal DNA isolation kit (MoBio, Carlsbad, CA, USA)	Real-time PCR	93.8% (n=13)	
Cryptosporidium hominis	ZR Fecal DNA MiniPrep™ Kit (Zymo Research, Irvine, CA)	Real-time PCR	100% (n=12)	[19]
Cryptosporidium parvum	ZR Fecal DNA MiniPrep™ Kit (Zymo Research, Irvine, CA)	Real-time PCR	100% (n=11)	
Cryptosporidium felis	ZR Fecal DNA MiniPrep™ Kit (Zymo Research, Irvine, CA)	Real-time PCR	100% (n=3)	
Blastocystis sp.	ZR Fecal DNA MiniPrep™ Kit (Zymo Research, Irvine, CA)	PCR	94% (n=50)	[20]
Enterocytozoon bienuesi	QIAamp stool mini kit (Qiagen Hilden, Germany)	PCR	100% (n=290)	[21]
Toxoplasma gondi	RTA DNA Isolation Kit from stool (Istambul, Turkey)	LAMP	100% (n=41)	[22]
